# Semblans: automated assembly and processing of RNA-seq data

**DOI:** 10.1093/bioinformatics/btaf003

**Published:** 2025-01-09

**Authors:** Miles D Woodcock-Girard, Eric C Bretz, Holly M Robertson, Karolis Ramanauskas, Jarrad T Hampton-Marcell, Joseph F Walker

**Affiliations:** Department of Biological Sciences, University of Illinois at Chicago, Chicago, IL 60607, United States; Department of Biological Sciences, University of Illinois at Chicago, Chicago, IL 60607, United States; The Sainsbury Laboratory, University of Cambridge, Cambridge, CB2 1LR, United Kingdom; Department of Genetics, University of Cambridge, Cambridge, CB2 3EJ, United Kingdom; Department of Biological Sciences, University of Illinois at Chicago, Chicago, IL 60607, United States; Department of Biological Sciences, University of Illinois at Chicago, Chicago, IL 60607, United States; Department of Biological Sciences, University of Illinois at Chicago, Chicago, IL 60607, United States

## Abstract

**Motivation:**

Recent advancements in parallel sequencing methods have precipitated a surge in publicly available short-read sequence data. This has encouraged the development of novel computational tools for the *de novo* assembly of transcriptomes from RNA-seq data. Despite the availability of these tools, performing an end-to-end transcriptome assembly remains a programmatically involved task necessitating familiarity with best practices. Aside from quality control steps, including error correction, adapter trimming, and chimera filtration needing to be correctly used, moving data between programs often requires manual reformatting or restructuring, which can further impede throughput. Here, we introduce *Semblans*, a tool for streamlining the assembly process that efficiently and consistently produces high-quality transcriptome assemblies.

**Results:**

*Semblans* abstracts the key quality control, reconstitution, and postprocessing steps of transcriptome assembly from raw short-read sequences to annotated coding sequences. Evaluating its performance against previously assembled transcriptomes on the basis of assembly quality, we find that *Semblans* produced higher quality assemblies for 98 of the 101 short-read runs tested.

**Availability and implementation:**

*Semblans* is written in C++ and runs on Unix-compliant operating systems. Source code, documentation, and compiled binaries are hosted under the GNU General Public License at https://github.com/gladshire/Semblans.

## 1 Introduction

Breakthroughs in massively parallel sequencing technologies have made sequence analysis far more accessible to researchers, leading to a wealth of publicly available sequence data. Sequencing the transcribed genomic regions (transcriptomic sequencing) is a cost-effective approach to obtaining protein-coding regions. Powerful new tools for RNA-seq data processing and assembly have been developed to facilitate this, enabling cost-effective avenues for RNA-seq analysis and retrieval of genomic coding regions.

Best practices dictate that reads typically undergo several steps of preprocessing and quality control, including correcting sequencing errors, trimming synthetic adapter sequences, removing foreign sequence contaminants, and filtering overrepresented reads. Assembly tools produce contigs from the cleaned reads, which introduce assembly artifacts. To alleviate the propagation of these errors, following assembly, several additional rounds of processing, such as filtering chimeric transcripts, removing poorly supported transcripts, and predicting coding regions, further improve the integrity and accuracy of the transcriptome assembly. Each phase of the data preparation process calls for specialized bioinformatics techniques and software tools, which must be used in the correct order and with the appropriate settings to avoid the proliferation of downstream errors. Several pipelines have been developed that use external tools to handle these steps for the user. For information on these pipelines and the tools they integrate, we refer the reader to Table 1 in Fallon *et al.* (2023). However, many of these pipelines’ assembly processes end with the *de novo* assembly of contigs and often do not take measures to identify and remove artifacts introduced during assembly.

The present work introduces *Semblans* (pronounced “semblance”), which builds upon previous work (Yang and Smith 2014, MacManes 2018, Rey *et al.* 2019, Rivera-Vicéns *et al.* 2022, Fallon *et al.* 2023, Ramanauskas and Igić 2023) by optimizing these intermediary steps at a lower programmatic level. A common hindrance to speed and scalability comes from the reformatting of large sequence data between successive program calls along the transcriptome assembly process. *Semblans* leverages the data streaming capabilities of C++ to enable the high-performance processing of data from one program to the next. *Semblans* has been tested across a variety of different computing architectures, including the NSF-funded cyberinfrastructure ecosystem (NSF ACCESS) (Boerner *et al.* 2023). We demonstrate that revisiting previous assemblies with *Semblans* can improve the quality of the assembly, generating a higher-quality transcriptome. *Semblans* allows for granular customization of its programs’ settings by the user through an included configuration file or with minimal configuration, using empirically tested default settings with versatility in mind to offer largely code-free execution without sacrificing user flexibility.

## 2 Workflow

**Figure 1.**
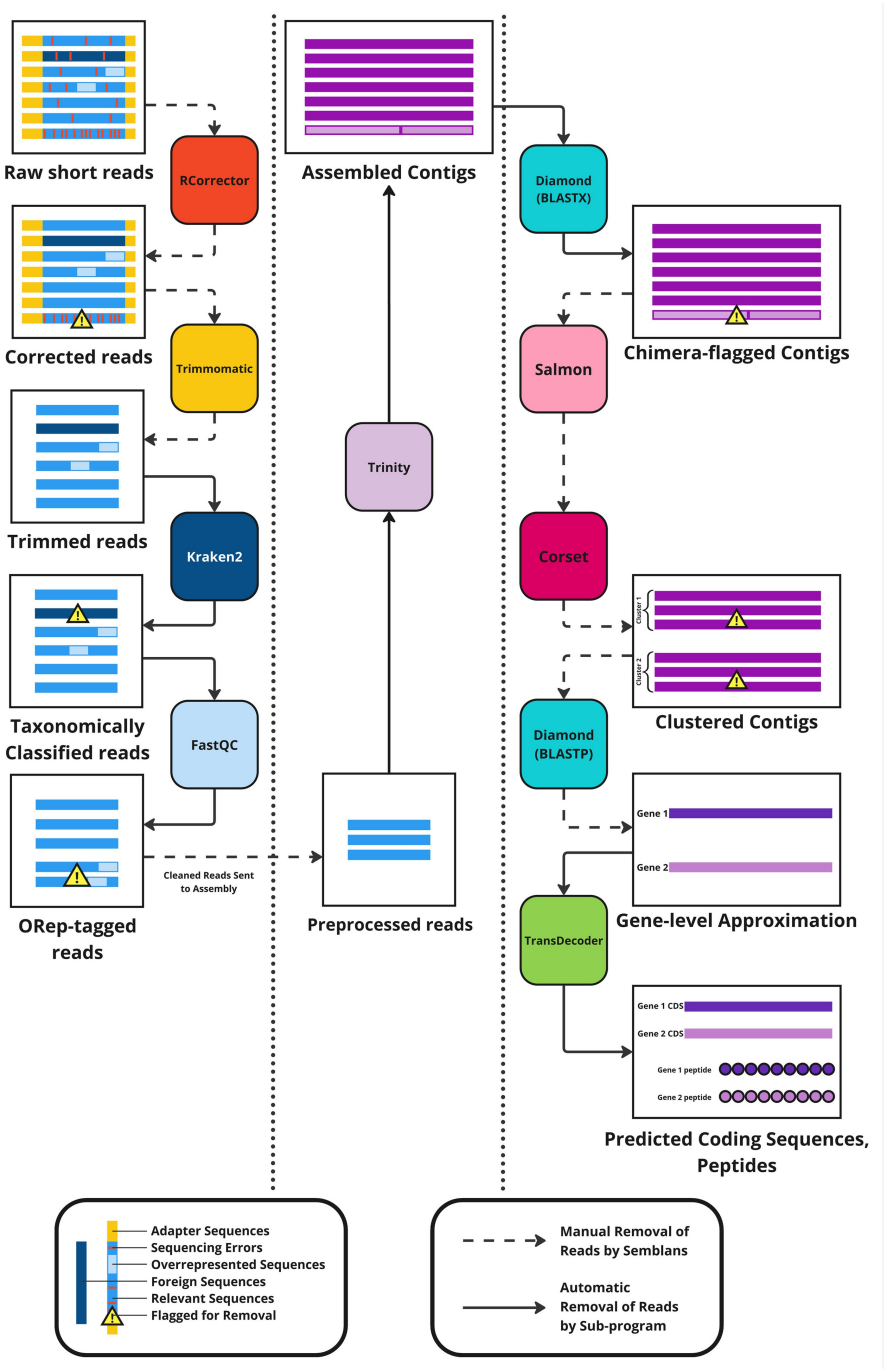
Workflow diagram of *Semblans*, demarcated according to its three successive phases: preprocess (left), assembly (middle), and postprocess (right). Also highlighted are the error type and the program or approach utilized to remove it. Not shown is the user-defined reference proteome, which informs the BLASTX- and BLASTP-compatible alignments during the postprocess phase.

*Semblans* is implemented in three successive phases, which can be run individually or in tandem: the cleaning of short-reads (preprocessing), the construction of cleaned reads into contigs (assembly), and the further refinement of these contigs (postprocessing). All steps within each phase of *Semblans*, save for an initial *FastQC* (https://qubeshub.org/resources/fastqc) quality analysis during the preprocessing phase, assembly via *Trinity* (Grabherr *et al.* 2011) during the assembly phase, and the final prediction of coding regions and peptides via *TransDecoder* (https://github.com/TransDecoder/TransDecoder) during the postprocessing phase, are optional and may be omitted at the user’s discretion. Figure 1 provides a graphical summary of the *Semblans* workflow.

### 2.1 Preprocess

During the *Semblans* preprocessing phase, reads undergo a preliminary quality screening by *FastQC*. *Semblans* then corrects single-base sequencing errors using the *k*-spectrum-based method implemented by *Rcorrector* (Song and Florea 2015). Errors deemed “unfixable” by *Rcorrector* are then removed by *Semblans*. If synthetic adapter sequences were detected during the initial quality analysis run, *Semblans* will call on *Trimmomatic* (Bolger *et al.* 2014) to clean them from the reads. *Semblans* will then perform a taxonomy-based refinement of reads using the *k*-mer-based taxonomic classification of foreign reads implemented by *Kraken2* (Wood *et al.* 2019). Reads are deemed “foreign” if they are successfully classified against a *Kraken2* database and are then removed from the set. As a final preprocessing step, *Semblans* removes overrepresented sequences (comprising >0.1% of sequence data) that are identified by a second *FastQC* quality analysis.

### 2.2 Assembly

After preprocessing, *Semblans*’ assembly phase uses *Trinity* to assemble transcripts *de novo* from the cleaned short-reads, generating a preliminary set of contigs for downstream processing.

### 2.3 Postprocess

Following the assembly phase, *Semblans* performs several postprocessing steps to refine assembly quality. Existing assembly pipelines implement rigorous preprocessing steps as outlined in Fallon *et al.* (2023), but few implement further postprocessing steps to address the errors known to arise with *de novo* assembly tools. *Semblans* includes approaches that detect and remove these commonly encountered assembly artifacts. First, contigs erroneously assembled from multiple transcripts, known as “chimeras,” are detected through a BLASTX-compatible alignment to a user-provided set of reference peptides using the *Diamond* (Buchfink *et al.* 2015) software package. *Semblans* then removes them according to the procedure in Yang and Smith (2013). *Salmon* (Patro *et al.* 2017) is then used to quantify read support for the assembled transcripts. These results guide *Semblans*’ subsequent use of *Corset* (Davidson and Oshlack 2014) to cluster transcripts. *Semblans* then extracts a gene-level approximation of the sequence data from the *Corset* output. Then, *Semblans* performs a BLASTP-compatible *Diamond* alignment of the transcripts against the same reference proteome as before, the results of which are leveraged to predict and report a final set of coding regions and peptides using the *TransDecoder* package. Finally, the transcripts may be annotated using *HMMER* (Eddy 2011) against the *PANTHER* functional peptide (Thomas *et al.* 2003) database.

## 3 Use case

**Figure 2.**
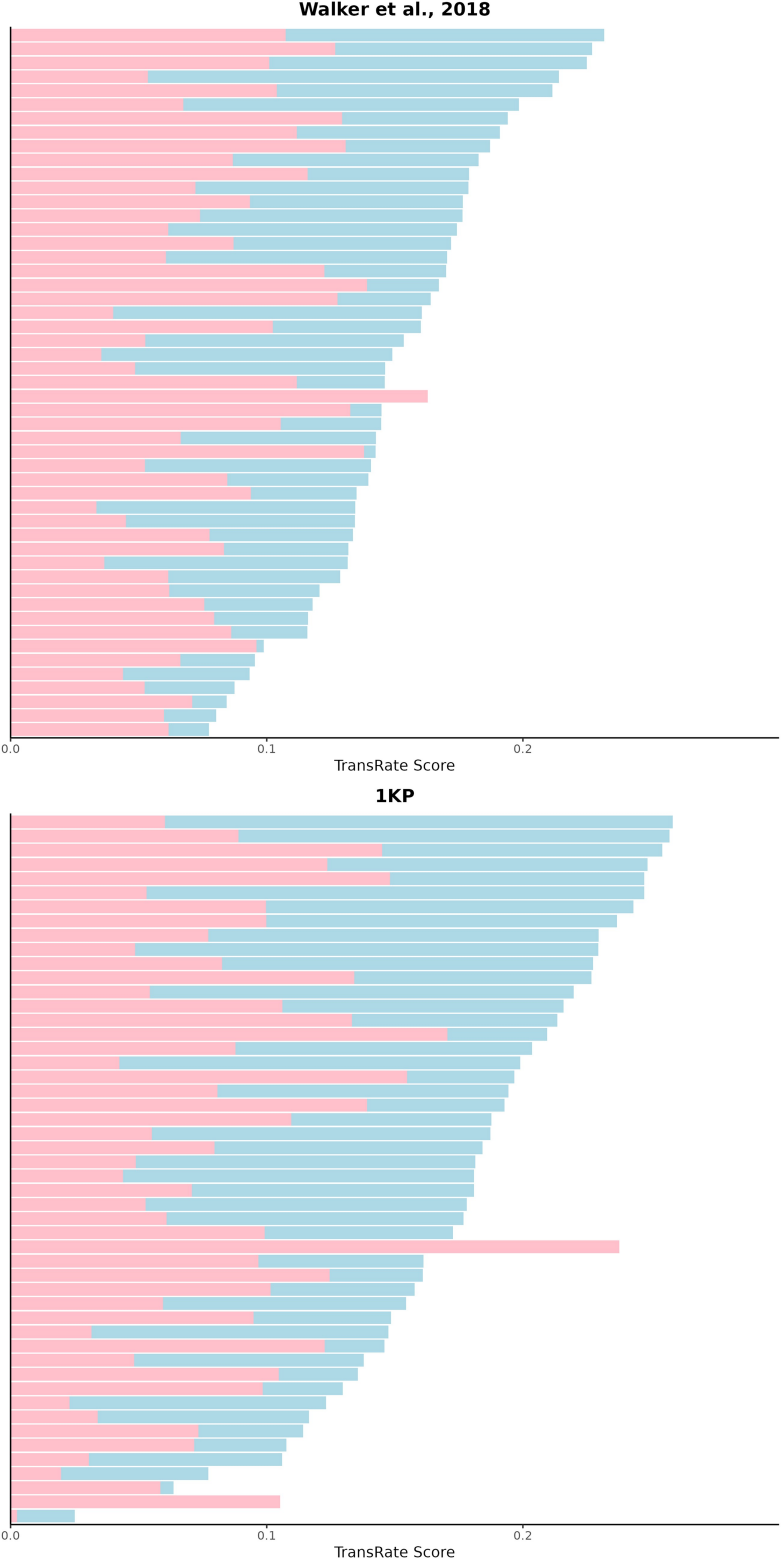
Comparison of the *TransRate* assembly scores for Caryophyllales (top) and 1KP (bottom) data between assembly approaches. Assemblies are sorted according to descending *Semblans* assembly score. Transcriptomes assembled with *Semblans* (blue) achieve consistently higher *TransRate* scores than those assembled by other approaches (red).

To assess assembly quality and the value of revisiting previous assemblies, we retrieved the raw sequence reads from 101 previously published transcriptomes and reassembled them using *Semblans*. Figure 2 compares the resulting qualities of these newer assemblies to those of the assemblies from the original studies. Data from two major sequencing projects were revisited: 50 samples from the 1KP (1000 plant transcriptomes) project that broadly sampled the plant tree of life (Zuntini *et al.* 2019) and another 51 samples from a project focused on the plant order Caryophyllales (Walker *et al.* 2018).

Quality was assessed using *TransRate* v.1.0.3 (Smith-Unna *et al.* 2016) by mapping the processed sequencing reads to the assemblies. The *TransRate* score penalizes assemblies based upon the number of predicted chimeric sequences, the degree of coverage provided for predicted transcripts, the number of SNPs between the mapped reads and the assembled transcripts, and how often both reads map to the same transcript. We found that *Semblans* produced higher-quality assemblies for 50 of the 51 Caryophyllales transcriptomes and 48 of the 50 1KP transcriptomes, demonstrating that revisiting prior sequencing data with *Semblans* can improve transcriptome quality. Supplementary Tables S1–S4 outline all assembly scores as well as the metrics composing them.

## 4 Future work

Although all steps of *Semblans* are abstracted from the user, its source code has been written with modularity in mind so as to ensure ease of maintenance and relevance as its dependencies are updated or as novel computational tools are introduced. In alignment with our goal of facilitating transcriptome assembly, we anticipate that updates to *Semblans* will follow this release.

## 5 Conclusion

The present work introduces *Semblans*, a flexible automated program for streamlining transcriptome assembly. By unifying cutting-edge bioinformatics tools under a single interface, *Semblans* builds off the frameworks established by previous programs to offer a user-friendly, scalable approach to transcriptome assembly.

## Supplementary Material

btaf003_Supplementary_Data

## Data Availability

The software, documentation and examples outlining the software’s functionality can be found at https://github.com/gladshire/Semblans.
